# Characterization of spatiotemporal and kinetic gait variables in dogs with hindlimb ataxia and bilateral hindlimb lameness

**DOI:** 10.1186/s12917-024-04265-8

**Published:** 2024-09-11

**Authors:** Clair Park, Dominique M. Sawyere, Theresa E. Pancotto, Otto I. Lanz, Stephen R. Werre

**Affiliations:** 1 Department of Small Animal Clinical Sciences, Virginia-Maryland College of Veterinary Medicine, Blacksburg, VA 24061 USA; 2 Metropolitan Veterinary Associates, Norristown, PA 19403 USA; 3 Specialists in Companion Animal Neurology, Naples, FL 34119 USA; 4 Laboratory for Study Design and Statistical Analysis, Virginia-Maryland College of Veterinary Medicine, Blacksburg, VA 24061 USA

## Abstract

**Background:**

Discriminating the underlying cause of gait abnormalities can be challenging in a clinical setting, especially in the presence of bilateral disease. Pressure-sensitive walkways (PSWs) have been utilized to characterize the gait of dogs with various neurologic or orthopaedic conditions. The potential use of the PSW includes the discrimination of conditions that can be similar in clinical presentation, such as bilateral hindlimb lameness and hindlimb ataxia. The primary aim of this study was to describe the spatial, temporal, and kinetic gait parameters of dogs with hindlimb ataxia or bilateral hindlimb lameness and compare them to those of normal dogs. Forty-six dogs were prospectively recruited. The normal group included 20 dogs with normal neurologic and orthopaedic exams. The orthopaedic group included 15 dogs with bilateral hindlimb orthopaedic diseases with weight-bearing hindlimb lameness and normal neurologic exams. The neurologic group included 11 dogs with ambulatory paraparesis and normal orthopaedic exams. Each dog was walked across the PSW, and at least 3 valid trials were collected. The stride time, stance time, swing time, stride length, gait velocity, peak vertical force (PVF), vertical impulse (VI), and limb symmetry were recorded. The mean values of all parameters from the valid trials were calculated and used for data analysis. The outcomes were compared among all groups.

**Results:**

Compared with the normal group, the orthopaedic group had a significantly greater percent body weight distribution (%BWD) and vertical impulse distribution (VID) in the forelimbs. When comparing the spatiotemporal parameters, the neurologic group showed an increase in forelimb stance time compared to that of the normal group. Compared with that in the normal group, the stride velocity in the forelimbs in the orthopaedic group was greater. There were no significant differences in the kinetic parameters between the neurologic group and the normal group, nor in stride time or stride length among the groups.

**Conclusion:**

The gait parameters obtained by PSW demonstrated that the orthopaedic and neurologic groups may have different compensatory mechanisms for their gait deficiencies. These parameters can potentially be used to construct a predictive model to evaluate PSW as a diagnostic tool in future studies.

**Supplementary Information:**

The online version contains supplementary material available at 10.1186/s12917-024-04265-8.

## Background

Gait abnormalities in veterinary patients can be caused by orthopaedic or neurologic diseases, for which a thorough gait assessment and additional diagnostics are often required to determine the underlying cause [1, 2]. While visual assessment of veterinary patients is a critical part of the diagnostic process in these patients, it is a subjective evaluation by clinicians [3, 4]. Subjective evaluation has the advantage of being performed without the need for any specialized equipment. However, the subjective orthopaedic gait assessment depends on the skill level of the observer and is subject to interobserver variability, especially when the abnormality is subtle or bilateral [5–7]. Previous studies have demonstrated a poor correlation between the subjective gait scoring system and force plate gait analysis in dogs with lameness, further limiting the role of subjective evaluation as a diagnostic tool [6, 7].

Objective gait assessments can be provided by force plate analysis, pressure-sensitive walkways (PSW), and stance analysers [8–10]. The PSW is a relatively new technology developed to analyse gait symmetry and can provide an objective measure of spatiotemporal gait parameters. These parameters include stride time, stride length, stance time, swing time, velocity, and calculated kinetic parameters such as peak vertical force (PVF) and vertical impulse (VI). While force plate analysis is often considered the ‘gold standard’ for objective gait analysis, PSW has been shown to generate consistent and precise gait data and has been validated as a reliable alternative method for assessing kinetic gait parameters in dogs [8, 11–13]. The advantages of the PSW over force plate analysis are that the PSW can characterize and evaluate spatiotemporal gait parameters and provide consecutive measurements from all limbs simultaneously over multiple gait cycles in normal dogs [14, 15]. Gait parameters obtained by the PSW are frequently used to characterize the gait of humans and animals with various orthopaedic or neurologic conditions for diagnostic and monitoring purposes [16–19]. Another potential use of the additional gait parameters obtained from the PSW might be to serve as a diagnostic tool to discriminate conditions that can be similar in clinical presentation, such as hindlimb ataxia and bilateral hindlimb lameness, in dogs.

Several canine studies have investigated spatiotemporal and kinetic parameters in dogs with neurologic or orthopaedic conditions using the PSW [20–24]. However, to the best of the authors’ knowledge, there are no studies comparing both kinetic and spatiotemporal gait parameters among clinically normal dogs, dogs with bilateral hindlimb lameness, and dogs with hindlimb ataxia. Therefore, the aim of this study was to describe the spatial, temporal, and kinetic gait parameters of dogs with hindlimb ataxia or bilateral hindlimb lameness to determine whether there were any discriminating variables in each group compared to those of normal group. We hypothesized that the PSW can be used to characterize distinct changes in the gait parameters of dogs with hindlimb ataxia and bilateral hindlimb lameness compared to those of clinically normal dogs and potentially identify gait parameters that can be used to differentiate the two gait patterns from the normal gait.

## Methods

This study was conducted with the approval of the Institutional Animal Care and Use Committee of Virginia Tech (IACUC protocol #19–146), and a signed owner consent form was obtained for each dog. Client-owned dogs were prospectively recruited at the Virginia-Maryland College of Veterinary Medicine Teaching Hospital from 2019 to 2022. Dogs over 1 year of age, weighing between 4.5 and 60 kg, were enrolled in the study. The lower weight limit was determined according to the manufacturer’s recommendation for ensuring reliable detection of individual foot strikes by the equipment. A power analysis using PASS 16 (Power Analysis and Sample Size Software (2018). NCSS, LLC. Kaysville, Utah, USA) showed that 15 dogs per group would be needed to detect a difference with a power of 80%. As a prospective pilot study, the maximum number of subjects enrolled within the study period were included in each group. Each dog enrolled in this study underwent an orthopaedic and neurologic examination, which was performed by a board-certified surgeon and neurologist or house officers under the supervision of a board-certified specialist who were blinded to the group assignment. Each dog was assigned a lameness score for each individual limb based on a subjective five-point grading scale, as shown in Table 1 [25]. A Modified Frankel Scale was used to assess the neurologic status of the dogs, as shown in Table 2 [26]. The group assignment and exam findings of each dog were recorded by one of the investigators (CP).

**Table 1:** The numerical rating score system used for subjective gait assessment

**Grade 0**	No lameness
**Grade 1**	Subtle lameness is present but inconsistent; apparent only at trot
**Grade 2**	Mild weight-bearing lameness obviously present at walk
**Grade 3**	Moderate weight-bearing lameness; lameness at work or trot
**Grade 4**	Non-weight-bearing lameness

**Table 2:** Modified Frankel Scale

**Grade 1**	Normal gait with paraspinal hyperesthesia
**Grade 2**	Ambulatory paraparesis
**Grade 3**	Nonambulatory paraparesis
**Grade 4**	Paraplegia with intact pain perception
**Grade 5**	Paraplegia with absent deep pain perception

The inclusion criteria for the normal group were dogs with normal orthopaedic and neurologic examination results performed by board-certified surgeons and neurologists and no history of orthopaedic or neurologic disease or any other significant comorbidities. The inclusion criteria for the neurologic group were dogs with ambulatory paraparesis (Modified Frankel Scale grade 2) with pelvic limb proprioceptive ataxia and a normal orthopaedic examination. Dogs were included if they were diagnosed with neurologic diseases causing a thoracolumbar myelopathy, based on advanced imaging findings, diagnostic results, and neurologic examination. Dogs were excluded if they had neurologic abnormalities in the forelimbs or if they had a history of orthopaedic disease or orthopaedic surgery. The inclusion criteria for the orthopaedic group were dogs with orthopaedic examination findings consistent with bilateral hindlimb orthopaedic diseases that exhibited weight-bearing hindlimb lameness (grades 1–3) and a normal neurologic examination. Dogs were excluded if they had forelimb gait abnormalities, a history of neurologic disease or neurologic surgery.

### Data collection

The data were collected using a 1.95 m × 0.45 m PSW (Walkway High-Resolution; Tekscan Inc., South Boston, Massachusetts, USA). The PSW was calibrated to the weight of the dogs as directed by the manufacturer’s guidance before each use. Designated software (Walkway 7.80x software; Tekscan Inc., South Boston, Massachusetts, USA) was used for data acquisition and analysis. Dogs were walked by one handler on a loose neck lead and were acclimated to the walkway first by walking the dog for 3 to 5 min across the mat. A trial was considered valid if the dog walked in a straight line along the entire length of the PSW, was not noticeably distracted, each foot strike remained within the pressure mat, the velocity was maintained between 0.8 m/s and 1.4 m/s, and the acceleration was between − 0.5 m/s^2^ and 0.5 m/s^2^. After accommodation, at least 3 valid trials were recorded for each dog. A video was recorded for each trial for review. All data acquisition was monitored and recorded by the investigator (CP) who was not blinded to the group assignment.

### Data processing

**Fig. 1:**
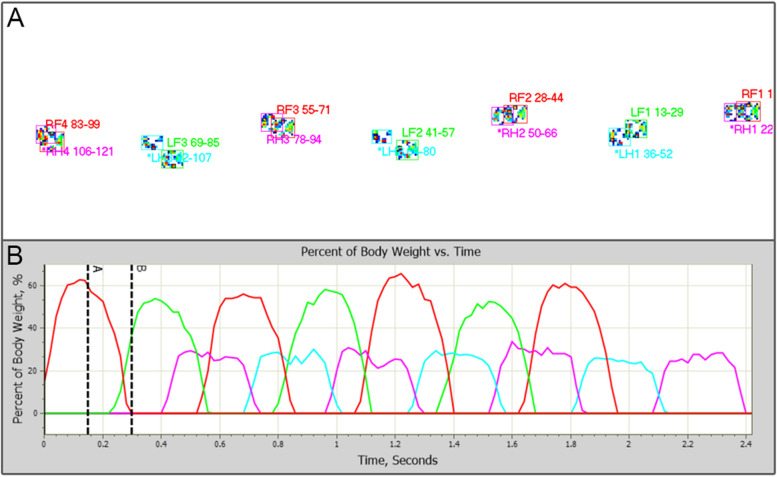
Representative data acquired from a subject in the normal group. **A** A footstrike recording of the subject. **B** Graph of percent body weight (%BW) over time generated from the data

After data acquisition, the custom software automatically identified the foot strikes and assigned LF (left forelimb), RF (right forelimb), LH (left hindlimb), or RH (right hindlimb) accordingly (Fig. 1). All videos of the trials were reviewed to ensure that each foot strike was correctly identified and assigned manually when needed. The mean values of all parameters from the minimum of 3 valid trials were calculated and used for data analysis. The spatial and temporal gait variables included stride time (seconds, s), stance time (seconds, s), swing time (seconds, s), stride length (meters, m), and gait velocity (meters per second, m/s). The kinetic variables included PVF (Newtons, N) and VI (Newton seconds, Nˑs). The PVF and VI values were normalized to body weight and were represented as a percentage of body weight distribution (%BWD) and VI distribution (VID), respectively, as previously described [9, 27]. Additionally, the limb symmetry between the forelimbs and hindlimbs was calculated using the following formula as previously described [28].

$$SI=\left(\frac{\left[\mathrm{XRF}+\mathrm{XLF}\right]-\left(\mathrm{XRH}+\mathrm{XLH}\right)}{0.5\ast\left(\mathrm{XRF}+\mathrm{XLF}+\mathrm{XRH}+\mathrm{XLH}\right)}\right)\times100$$

RF = right forelimb, LF = left forelimb, RH = right hindlimb, LH = left hindlimb

An SI = 0 indicates complete symmetry, an SI > 0 indicates that the forelimbs have higher values, and an SI < 0 indicates that the hindlimbs have higher values.

Asymmetry between the right and left limbs was calculated using the following formula.

$$SI=\left(\frac{\left[\mathrm{XRF}-\mathrm{XLF}\right]}{0.5\ast\left(\mathrm{XRF}+\mathrm{XLF}\right)}\right)\times100$$

An SI = 0 indicates complete symmetry, an SI > 0 indicates that the right limbs had a higher value and SI < 0 indicates that the left limbs had a higher value. The same formula was used for SI of right and left hindlimbs.

### Statistical analyses

The normality of the data was determined by generating and inspecting a normal probability plot. The normal probability plots showed that all numerical variables, including weight, age, and gait parameter outcomes, were skewed. Accordingly, kinetic and temporospatial values were expressed as median, minimum, and maximum. The outcomes of the gait analyses were compared between groups using the Kruskal‒Wallis test followed by Dunn’s test for multiple comparisons. The association between sex and group was assessed using Fisher’s exact test. Overall, *p* values were adjusted for multiple testing using the Benjamini–Hochberg false discovery rate method. Statistical significance was set to *P* < 0.05. All analyses were performed using SAS version 9.4 (Cary, North Carolina, USA).

## Results

Forty-six dogs were enrolled in the study. The normal group included 20 dogs, 12 neutered males, and 8 spayed females, with a median age of 6 years (range, 1–12 years) and a median weight of 22.3 kg (range, 7.6–40.5 kg). The dog breeds included mixed-breed (*n* = 14), Walker Hound (2), and one each of the following breeds: Border Collie, Danish Swedish Farm dog, Labrador Retriever, and Staffordshire Terrier. The orthopaedic group included 15 dogs, 7 neutered males, and 8 spayed females with a median age of 6 years (range, 2–12 years) and a median weight of 30.0 kg (range, 17–59 kg). The dog breeds included mixed-breed dog (*n* = 5), Labrador Retriever (2), Staffordshire Terrier (2), and one each of the following breeds: Mastiff, English Bulldog, German Shepherd, American Foxhound, Siberian Husky, and English Springer Spaniel. The most common diagnoses attributable to bilateral lameness in the group included cranial cruciate ligament disease (*n* = 7), followed by hip dysplasia (5), grade III/IV medial patella luxation (2), and chronic iliopsoas pain (1). The neurologic group included 11 dogs, 5 neutered males, and 6 spayed females, with a median age of 8 years (range, 4–11 years) and a median weight of 16.5 kg (range, 4.8–52.0 kg). The dog breeds were mixed breed (*n* = 4), Dachshund (3), and one each of the following breeds: Bull Mastiff, English Bulldog, Brittany Spaniel, Boxer, or Shih Tzu. The prevalent diagnoses included intervertebral disc extrusion or protrusion (*n* = 9), acute non-compressive nucleus pulposus extrusion (1), and degenerative myelopathy (1). There was no significant difference in age or sex among the groups. Overall, the average weight of the orthopaedic group was greater than that of the normal group and the neurologic group (*p* < 0.01). There was no significant difference between the average weight of the normal group and the neurologic group (*p* = 0.57).

The normalized kinetic gait parameters are summarized in Table 3. Compared with the normal group, the orthopaedic group had greater %BWD and VID in the forelimbs and lower values in the hindlimbs. The differences were statistically significant (*p* < 0.001) for all the individual limbs except for the left hindlimb, which closely approached statistical significance (*p* = 0.051). The SI of the maximum force between the right hindlimb and the left hind limb in the orthopaedic group was significantly lower than that in the normal group (*p* = 0.005). There were no significant differences in the %BWD or VID between the normal group and the neurologic group or between the orthopaedic group and the neurologic group.

**Table 3:** Comparison of the kinetic parameters normalized to body weight among the normal, orthopaedic, and neurologic groups

	Groups		
	Normal	Orthopaedic	Neurologic
PVF(%BWD)			
LF	28.30 (27.85–30.25)	32.00 (30.20–33.90)	29.60 (27.50–33.40)
RF	29.00 (27.20–30.30)	32.60 (30.30–40.80)	29.20 (27.50–32.40)
LH	21.60 (20.30–23.10)	18.10 (15.20–22.60)	20.50 (17.10–22.30)
RH	20.65 (19.45–21.90)	17.80 (16.30–19.20)	19.10 (16.70–23.50)
VID(%BW*s)			
LF	30.15 (29.50-31.25)	33.30 (28.90–35.90)	31.60 (28.10–37.20)
RF	30.40 (28.90-31.25)	32.70 (30.20–37.00)	29.60 (28.30–34.90)
LH	19.65 (18.75–21.20)	17.60 (14.30–22.60)	19.60 (14.60–22.40)
RH	18.90 (17.75–20.30)	16.20 (13.50–18.50)	18.10 (14.90–22.30)

All values are expressed as medians (range)

*PVF* peak vertical force, *%BWD* body weight distribution, *VID* vertical impulse distribution, *LF* left forelimb, *RF* right forelimb, *LH* left hindlimb, *RH* right hindlimb

The SIs between the gait parameters of the forelimbs and the hindlimbs are reported in Table 4. The orthopaedic group had a greater forelimb: hindlimb SI of the maximum force (*p* = 0.0002) compared to those of the normal group. When comparing the SIs of the spatiotemporal gait parameters among the groups, the neurologic group had a greater forelimb: hindlimb stance time compared to the normal group (*p* = 0.02). In contrast, there was no significant difference in the stance time between the orthopaedic group and the normal group (*p* = 0.148). The neurologic group had the lowest SIs of stride time and stride length in the forelimbs; however, the differences in the values among the groups were not statistically significant (*p* > 0.07). The orthopaedic group had a greater stride velocity in the forelimbs than in the hindlimbs, as demonstrated by the significantly greater SI value in the orthopaedic group than in the normal group (*p* = 0.009). There was no significant difference in the SI of the stride velocity between the neurologic group and the normal group (*p* = 0.36).

**Table 4:** Comparison of the SI of the forelimb and hindlimbs between the normal, orthopaedic, and neurologic groups

	Groups		
Forelimb: Hindlimb SI (%)	Normal	Orthopaedic	Neurologic
Stance Time	5.65 (3.39–9.32)^b^	13.08 (5.22–17.33)	16.70 (6.96–35.19)^b^
Stride Time	2.20 (-1.07-3.14)	2.50 (1.03–7.67)	1.61 (-42.62-6.92)
Stride Length	1.22 (0.43–2.23)	2.55 (0.75–13.04)	1.13 (-27.42-5.55)
Stride Velocity	2.58 (1.54–4.69)^a^	5.21 (3.21–8.62)^a^	2.61 (1.45–12.40)
Maximum Force	30.95(20.02–39.10)^a^	58.87 (53.16–67.96)^a^	48.79 (23.18–63.05)

^a^statistically significant difference between the normal group and the orthopaedic group

^b^statistically significant difference between the normal group and the neurologic group

^c^statistically significant difference between the orthopaedic group and the neurologic group

## Discussion

This study demonstrated that compared to the normal group, the orthopaedic group had increased body weight distribution and increased stride velocity in the forelimbs, while the neurologic group had increased stance time in the forelimbs without significant changes in body weight distribution or stride velocity. These results support our hypothesis that PSW could detect distinct changes in kinetic and spatiotemporal gait parameters in dogs with bilateral hindlimb lameness and in dogs with hindlimb ataxia when compared to the normal dogs. Additionally, the results also indicate that dogs with bilateral hindlimb lameness and hindlimb ataxia may compensate for their gait abnormalities through different mechanisms. While thorough neurologic and orthopaedic exams are pivotal for the differentiation of two presentations, PSW analysis may provide additional information for clinicians to help in the differentiation of neurologic and orthopaedic hind limb disease.

For the kinetic parameters, the orthopaedic group had significantly greater %BWD and VID in the forelimb, as well as greater forelimb: hindlimb SI maximum force, than did the normal group. This indicates that dogs with bilateral hindlimb orthopaedic disease compensate by shifting their weight to their forelimbs; dogs with hindlimb ataxia did not demonstrate this effect. A possible explanation for the difference in compensation is that orthopaedic diseases often accompany variable degrees of pain associated with osteoarthritis in the affected limb, which may be alleviated by a redistribution of the weight to the unaffected limbs. On the other hand, the gait abnormalities in dogs with paraparesis and ataxia do not always involve pain in the affected limbs. This “compensatory cranial weight shift” in dogs with bilateral hindlimb orthopaedic diseases is commonly described in the clinical setting, however, objective data based on comparisons with a control group are sparse [29, 30]. In a healthy dog, weight is approximately distributed 60% to the forelimbs and 40% to the hindlimbs, regardless of the body weight or size [29, 31–33]. The median body weight distributions of the normal group in the current study were 28.3% (range: 25.9–32.9%) and 29.00% (25.0-33.1%) for the forelimbs and 21.6% (17.4–24.1%) and 20.65% (17.2–24.8%) for the left and right hindlimbs, respectively, which are comparable to the previously reported normal values for dogs and confirmed that the normal group was valid as a control.

Multiple previous studies evaluating the weight distribution of dogs with various naturally occurring or experimentally induced lameness have shown that dogs with lameness tend to redistribute their weight to the non-affected limbs, mainly to the contralateral and diagonal limbs, thus exhibiting more side-to-side compensation rather than a caudal-to-cranial shift [23, 32–35]. However, a recent study utilizing a stance analyser to evaluate weight-bearing compensation in police-working dogs with bilateral hip osteoarthritis revealed that affected dogs had weight shift to the thoracic limbs, corroborating the common clinical description of “cranial weight shift” in bilaterally affected dogs [36]. The results from the current study are not only in line with these findings but also demonstrate statistical differences in the kinetic parameters in comparison to those of a normal control group to provide further objective parameters in support of the commonly accepted cranial weight shift phenomenon in dogs with bilateral hindlimb lameness.

In contrast to the orthopaedic group, the neurologic group showed similar body weight distribution in all limbs compared to the normal group, indicating that compensatory mechanisms may be different in dogs with paraparesis and proprioceptive ataxia secondary to thoracolumbar myelopathy. Changes in kinetic parameters in dogs with various neurologic diseases have been investigated in previous studies [20, 37–39]. One study evaluated kinetic gait parameters in Doberman Pinchers with cervical spondylomyelopathy and found that there was no significant difference in the %BWD of the forelimb or the hindlimb compared to clinically normal dogs [20]. Another study revealed no significant differences in the PVF between the hindlimbs of normal Dachshunds and Dachshunds that underwent hemilaminectomy for thoracolumbar intervertebral disc disease [40]. This study also showed that the post-hemilaminectomy group had greater PVF on the more affected limb, which was contrary to findings in dogs affected by orthopaedic disease where lower PVF is expected in the limb with impaired function [5, 6, 23, 40, 41]. Along with the results from the current study, these findings support that dogs with ataxia may not necessarily redistribute their body weight to compensate for their uncoordinated gait. An alternative explanation is that these dogs might be unable to control the force exerted by the limbs due to loss of input from the upper motor neuron, preventing them from consciously compensating for the instability in their hindlimbs.

Interestingly, in another study that evaluated gait parameters in dogs with ataxia due to thoracolumbar myelopathy, the affected dogs had greater PVFs in the forelimbs than did the normal dogs [21]. The authors of that study postulated that dogs with thoracolumbar neurologic disease tend to shift their weight to the forelimbs as a result of hindlimb ataxia and instability [21]. This finding is in contrast with our data. The disparity in the observations between the studies may be due to several factors. In the aforementioned study, Dachshunds were overrepresented, composing 70% of the neurologic group in that study, while the neurologic group in our study included a more diverse population of dogs of various sizes, breeds, and conformations, with only 3 of 11 dogs being chondrodystrophic. Although the influences of the variation in the body weight and sizes of dogs can be avoided by the use of kinetic values normalized to the body weight, one study has suggested that there can be significant differences in the fully normalized ground reaction force and impulse distribution in the forelimb versus hindlimb between various dog breeds [27, 42]. Therefore, the overrepresentation of a certain breed or body conformation may have led to different results in the distribution of body weight [42]. Additionally, the difference in the velocity range of the subjects and the method of normalization of this value may have led to different outcomes. It is known that kinetic gait parameters can be dependent on the velocity and acceleration of patients [43, 44]. To avoid this influence, previous investigators have suggested maintaining the variables within a range during gait analysis [43, 44]. In our study, all dogs were walked at velocities between 0.8 m/s and 1.4 m/s, in accordance with protocols validated in previous studies [43, 44]. In contrast, the neurologic group in that study first walked at their preferred pace, which initially led to a significantly lower velocity than that of the clinically normal group [21]. Although the difference was not significant after the velocities of the groups were adjusted for the heights of the subjects by regression analysis, these overall differences in the data collection protocol and processing method may have contributed to the discrepancies in the gait variables in different studies.

Some studies found that the dogs with spinal cord injuries exhibit cranial shifts in the centre of pressure and the weight distribution measured by digital scales, compared to the healthy controls [45, 46]. While the results may appear to be contradictory to that of the current study, it is notable that the severity of the diseases of the study population was very different in these studies, as both studies only included non-ambulatory paraparetic dogs or paraplegic dogs with or without pain perception. In contrast, the current study limited the inclusion criteria to dogs with ambulatory paraparesis and excluded any non-ambulatory dog, as the aetiology for these patients should be apparent at the time of the diagnosis and the comparison would be less clinically relevant. These differences in the findings suggest that the severity of the disease might influences the compensation mechanism in dogs with neurologic disease.

One of the notable changes in the neurologic group was the increase in the stance time of the forelimbs. When compared within a group, all groups had positive forelimb: hindlimb SI of the stance time, indicating that dogs in our study had a longer stance phase in the forelimbs than in the hindlimbs, regardless of their group. This finding is in agreement with another study that demonstrated longer stance times on the forelimbs than on the hindlimbs in dogs at a walk [47]. When comparing between groups, the forelimb: hindlimb SI stance time of the neurologic group was significantly greater than that of the normal group, which was not observed in the orthopaedic group. Instead, the orthopaedic group had relatively greater stride velocity in the forelimbs than the hindlimbs compared to the normal dogs. These distinct changes in spatiotemporal gait parameters in the orthopaedic and neurologic groups are likely reflective of different compensatory mechanisms for hindlimb instability: dogs in the neurologic group may stabilize their unsteady gait in the hindlimbs by increasing the time that the forelimbs are in contact with the ground, while dogs in the orthopaedic group may take quicker strides in the forelimbs to afford the increased weight distribution.

In our study, we found no significant differences in forelimb: hindlimb SI of the stride time or stride length among the groups. The neurologic group was the only group with negative mean values, as well as the lowest median forelimb: hindlimb SI values for stride time and stride length, but the differences between the neurologic group and the other groups did not reach statistical significance. Although our study did not find statistically corroborating results, a published study reported decreased stride time and stride length in the forelimbs of dogs with thoracolumbar myelopathy and hindlimb ataxia [22]. It has been shown that dogs with neurologic disease have greater variances in the spatiotemporal gait parameters compared to clinically normal dogs [21]. A larger sample size with the variables normalized to the height of the subjects may be required to detect statistically significant differences. In addition, the onset, progression, and chronicity of diseases in the neurologic group were not specified in the current study. These factors may have affected the degree or pattern of gait compensation. Future studies focusing on a specific disease process with a similar chronicity, disease progression, or neurologic grading may lead to different results.

The overall changes observed in the neurologic group included an increase in forelimb stance time and a relatively lower stride time and length. These findings are similar to those of a previously reported gait analysis study in rats with experimentally induced spinal cord injuries, in which the rats had increased stance time and decreased stride length in the forelimbs [48]. On the other hand, the orthopaedic group had increased body weight distribution and stride velocity in the forelimbs compared to those on the hindlimbs, without any increase in stance time. Thus, this can be interpreted as dogs with bilateral hindlimb lameness compensate by shifting body weight cranially, which subsequently increases the stride velocity of the forelimb to support weight shift during each gait cycle. Based on these observations, gait parameters such as %BWD, VID, forelimb: hindlimb SI of stance time, and stride velocity are worthy of further investigation to evaluate their potential to discriminate between the two presentations. A further study that determines the cut-off value for each variable and receiver operating characteristic (ROC) curve may assess the accuracy of the subset of these parameters in discriminating dogs with bilateral hindlimb lameness and ataxia based on the data obtained by the PSW.

The main limitation of the study was the relatively small sample size, which may have led to a type II error. This was demonstrated by the apparent sidedness observed in the orthopaedic group, even though all dogs within the group were confirmed to have bilateral orthopaedic diseases and decreased weight-bearing in both hindlimbs, as confirmed by gait analysis. The small sample size may also have affected the gait parameters for the neurologic group, especially when they had the greatest variability within the dataset, with greater ranges in many of the analysed gait parameters. While all dogs included in the neurologic group were classified as grade 2 (ambulatory paraparesis) based on the Modified Frankel Scale, the clinical status of individuals varied greatly from mild to severe ataxia. This inherent variability of the data in subjects with neurologic disease has been noted in both humans and dogs, and the use of the coefficient of variation of variables, rather than individual mean values, has been advocated for these patients [22, 49, 50]. Because the current study focused on the characterization of the spatiotemporal and kinetic gait parameters obtained by the PSW for each group to provide a pilot data for further investigation in their diagnostic potentials, the coefficient of variation was not included in the variables. Further studies including larger sample sizes and three-way comparisons of the coefficient of variation of the neurologic group, the orthopaedic group, and the normal group may reveal further discriminating gait parameters and patterns among the groups. Larger sample sizes will also help to build reliable predictive models based on the discriminating gait parameters found in the current study, which can be used to evaluate the reliability of the PSW as a diagnostic tool.

Another limitation of the current study is that the dogs were assigned to pre-selected groups based on the clinician’s subjective evaluation. Although this study established baseline gait parameters for each group and demonstrated the potential use of the PSW, its effectiveness in distinguishing between the groups could not be evaluated with the current study design. Future research will be needed to further verify the feasibility and utility of the PSW as a diagnostic tool. Our study included a heterogeneous group of dogs of various breeds, weights, and body conformations. Although this is a more accurate and practical presentation of clinical settings, one study suggested that various breeds, body conformations, heights, and weights of dogs can affect spatiotemporal and kinetic gait parameters [42]. There were no significant differences in age or sex among the groups in the current study, but the mean weight of the orthopaedic group was greater than that of the other groups. This was reflective of the fact that many of the dogs within the group were large breeds that presented with bilateral cruciate ligament disease. While authors are aware of this potential source of bias, it also reflects patient demographics encountered in a clinical setting. Additionally, a recent study showed that the %BWD and most SI values have low variability in a heterogeneous dog group [27]. The SI value also has a benefit in that it eliminates interpatient variability, as the patient serves as its own control. Thus, we focused on the comparison of the normalized kinetic variables and SI values to minimize the potential influence from the breeds and confirmation by comparing the changes in variables within the patient.

In conclusion, the orthopaedic and neurologic groups exhibited distinct changes in spatiotemporal and kinetic gait parameters compared to those of normal dogs. The findings also suggest that dogs with hindlimb gait abnormalities may have different compensatory mechanisms for their gait deficiencies depending on whether their gait abnormalities are orthopaedic or neurologic in origin. Compared to those in the normal group, significant differences were found in gait parameters such as the %BWD and forelimb: hindlimb SI values of the stride velocity of the orthopaedic group and in the SI stance time of the neurologic group. In the future, a larger-scale study may help to determine the optimal cut-off value and build predictive models based on the discriminating gait parameters found in the current study. This may further support the utility of the PSW in differentiating between two presentations that are often difficult to determine clinically.

## Supplementary Information

Supplementary Material 1.

Supplementary Material 2.

## Data Availability

All data on the gait parameters that support the findings of this study are included within the manuscript and its supplementary Information files.

## Abbreviations

PVF: Peak vertical force
VI: Vertical impulse
PSW: Pressure sensitive walkway
%BWD: Percentage of body weight distribution
VID: Vertical impulse distribution
SI: Symmetry index
RF: Right forelimb
LF: Left forelimb
RH: Right hindlimb
LH: Left hindlimb
