# Supplementary material for: Characterization of spatiotemporal and kinetic gait variables in dogs with hindlimb ataxia and bilateral hindlimb lameness
Source: BMC Vet Res. 2024 Sep 11;20:405. doi: 10.1186/s12917-024-04265-8 (PMC11389471; doi:10.1186/s12917-024-04265-8)
Supplement: Supplementary file 1 — Supplementary Material 1. [file 12917_2024_4265_MOESM1_ESM.docx]

| **Outcome** | **Group** | **n** | **Median (Range)** | **P_Value** | **Adjusted P-Value** |
| --- | --- | --- | --- | --- | --- |
| Age | 1 | 20 | 6.0 (1.0-12.0) | 0.2531 | 0.2795 |
|  | 2 | 15 | 6.0 (2.0-12.0) |  |  |
|  | 3 | 8 | 8.0 (4.0-11.0) |  |  |
| BodyWeightDistributionLF | 1 | 20 | 28.3 (25.9-32.9) | 0.0434 | 0.0839 |
|  | 2 | 15 | 32.0 (25.0-50.6) |  |  |
|  | 3 | 11 | 29.6 (21.7-35.4) |  |  |
| BodyWeightDistributionLH | 1 | 20 | 21.6 (17.4-24.1) | 0.1368 | 0.1686 |
|  | 2 | 15 | 18.1 (9.0-32.8) |  |  |
|  | 3 | 11 | 20.5 (9.3-26.3) |  |  |
| BodyWeightDistributionRF | 1 | 20 | 29.0 (25.0-33.1) | 0.0043 | 0.0336 |
|  | 2 | 15 | 32.6 (21.1-51.4) |  |  |
|  | 3 | 11 | 29.2 (20.4-43.1) |  |  |
| BodyWeightDistributionRH | 1 | 20 | 20.7 (17.2-24.8) | 0.0058 | 0.0336 |
|  | 2 | 15 | 17.8 (8.9-23.9) |  |  |
|  | 3 | 11 | 19.1 (16.0-25.8) |  |  |
| CyclesMinute | 1 | 20 | 94.5 (77.0-162.0) | 0.0397 | 0.0839 |
|  | 2 | 15 | 86.0 (72.0-120.0) |  |  |
|  | 3 | 11 | 117.0 (80.0-158.0) |  |  |
| GaitCycleTimesec | 1 | 20 | 0.6 (0.4-0.8) | 0.0443 | 0.0839 |
|  | 2 | 15 | 0.7 (0.5-0.8) |  |  |
|  | 3 | 11 | 0.5 (0.4-0.8) |  |  |
| GaitDistanFrontcm | 1 | 20 | 160.6 (126.9-173.2) | 0.2686 | 0.2847 |
|  | 2 | 15 | 154.7 (140.8-304.8) |  |  |
|  | 3 | 11 | 164.1 (146.0-324.3) |  |  |
| GaitTime_Frontsec | 1 | 20 | 1.5 (1.1-1.7) | 0.3042 | 0.3161 |
|  | 2 | 15 | 1.4 (1.1-3.7) |  |  |
|  | 3 | 11 | 1.7 (1.1-3.4) |  |  |
| GaitVelocityFrontcmsec | 1 | 20 | 110.5 (100.6-125.0) | 0.2187 | 0.2520 |
|  | 2 | 15 | 111.0 (94.3-132.6) |  |  |
|  | 3 | 11 | 99.7 (54.1-144.5) |  |  |
| MaxForceFrontHind | 1 | 20 | 31.0 (8.8-61.8) | 0.0012 | 0.0305 |
|  | 2 | 15 | 58.9 (8.2-109.9) |  |  |
|  | 3 | 11 | 48.8 (11.1-97.9) |  |  |
| MaxForceLeftHindRightHind | 1 | 20 | 6.9 (-13.6-16.0) | 0.0207 | 0.0577 |
|  | 2 | 15 | 17.3 (-123.9-54.6) |  |  |
|  | 3 | 11 | 26.4 (-21.0-53.1) |  |  |
| MaximumPeakPressurekPaLF | 1 | 18 | 375.0 (123.0-589.0) | 0.0068 | 0.0336 |
|  | 2 | 14 | 245.0 (72.0-523.0) |  |  |
|  | 3 | 11 | 142.0 (78.0-350.0) |  |  |
| MaximumPeakPressurekPaLH | 1 | 18 | 281.0 (102.0-382.0) | 0.0039 | 0.0336 |
|  | 2 | 14 | 225.0 (71.0-350.0) |  |  |
|  | 3 | 11 | 92.0 (73.0-327.0) |  |  |
| MaximumPeakPressurekPaRF | 1 | 18 | 366.0 (144.0-626.0) | 0.0023 | 0.0305 |
|  | 2 | 14 | 266.5 (76.0-683.0) |  |  |
|  | 3 | 11 | 135.0 (71.0-305.0) |  |  |
| MaximumPeakPressurekPaRH | 1 | 18 | 285.0 (100.0-397.0) | 0.0023 | 0.0305 |
|  | 2 | 14 | 207.5 (49.0-407.0) |  |  |
|  | 3 | 11 | 102.0 (63.0-254.0) |  |  |
| PVFkgLF | 1 | 18 | 17.0 (3.8-24.9) | 0.0145 | 0.0433 |
|  | 2 | 14 | 13.2 (2.7-34.8) |  |  |
|  | 3 | 11 | 4.1 (0.9-24.7) |  |  |
| PVFkgLH | 1 | 18 | 12.0 (2.8-19.7) | 0.0054 | 0.0336 |
|  | 2 | 14 | 8.1 (2.3-16.4) |  |  |
|  | 3 | 11 | 3.1 (0.8-15.3) |  |  |
| PVFkgRF | 1 | 18 | 16.4 (4.2-24.7) | 0.0084 | 0.0342 |
|  | 2 | 14 | 14.8 (2.7-40.8) |  |  |
|  | 3 | 11 | 4.0 (1.2-21.6) |  |  |
| PVFkgRH | 1 | 18 | 12.5 (2.8-18.1) | 0.0071 | 0.0336 |
|  | 2 | 14 | 7.3 (0.8-16.2) |  |  |
|  | 3 | 11 | 2.6 (0.6-12.4) |  |  |
| StanceTimeFrontHind | 1 | 19 | 5.7 (-7.1-21.6) | 0.0700 | 0.1108 |
|  | 2 | 15 | 13.1 (-6.5-29.7) |  |  |
|  | 3 | 11 | 16.7 (-36.1-62.7) |  |  |
| StanceTimeLeftHind_RightHind | 1 | 19 | 3.3 (-6.6-16.9) | 0.1348 | 0.1686 |
|  | 2 | 15 | 3.6 (-45.3-40.0) |  |  |
|  | 3 | 11 | 6.2 (-28.7-23.6) |  |  |
| StanceTimeSecLH | 1 | 20 | 0.4 (0.1-0.5) | 0.1001 | 0.1381 |
|  | 2 | 15 | 0.4 (0.2-0.5) |  |  |
|  | 3 | 11 | 0.3 (0.1-0.5) |  |  |
| StanceTimeSecRF | 1 | 20 | 0.4 (0.2-0.5) | 0.0131 | 0.0433 |
|  | 2 | 15 | 0.4 (0.2-0.5) |  |  |
|  | 3 | 11 | 0.3 (0.1-0.4) |  |  |
| StanceTimeSecRH | 1 | 20 | 0.4 (0.1-0.5) | 0.2245 | 0.2532 |
|  | 2 | 15 | 0.3 (0.1-0.5) |  |  |
|  | 3 | 11 | 0.3 (0.1-0.5) |  |  |
| StancetimeSecLF | 1 | 20 | 0.4 (0.2-0.5) | 0.0316 | 0.0753 |
|  | 2 | 15 | 0.4 (0.2-0.5) |  |  |
|  | 3 | 11 | 0.3 (0.1-0.4) |  |  |
| StrideAccelerationCmSec2LF | 1 | 14 | 7.9 (2.2-35.8) | 0.0244 | 0.0623 |
|  | 2 | 12 | 18.0 (2.4-49.9) |  |  |
|  | 3 | 8 | 39.4 (0.0-129.8) |  |  |
| StrideAccelerationCmSec2LH | 1 | 14 | 13.6 (5.3-48.8) | 0.1688 | 0.2033 |
|  | 2 | 10 | 7.0 (1.0-35.9) |  |  |
|  | 3 | 9 | 20.9 (4.8-78.6) |  |  |
| StrideAccelerationCmSec2RF | 1 | 13 | 14.2 (0.0-28.9) | 0.0460 | 0.0841 |
|  | 2 | 9 | 9.0 (2.3-45.2) |  |  |
|  | 3 | 9 | 48.9 (9.3-245.7) |  |  |
| StrideAccelerationCmSec2RH | 1 | 13 | 7.8 (0.0-55.8) | 0.1042 | 0.1381 |
|  | 2 | 11 | 14.6 (0.9-95.6) |  |  |
|  | 3 | 7 | 27.4 (3.5-86.4) |  |  |
| StrideLengthFrontHind | 1 | 19 | 1.2 (-5.0-4.1) | 0.1293 | 0.1671 |
|  | 2 | 15 | 2.6 (0.0-27.6) |  |  |
|  | 3 | 11 | 1.1 (-36.5-33.6) |  |  |
| StrideLengthLeftHindRightHind | 1 | 20 | 1.7 (-3.0-10.0) | 0.0432 | 0.0839 |
|  | 2 | 15 | 1.7 (-23.8-13.7) |  |  |
|  | 3 | 11 | 7.6 (-39.2-27.7) |  |  |
| StrideTimeFrontHind | 1 | 20 | 2.2 (-9.2-4.3) | 0.1893 | 0.2230 |
|  | 2 | 15 | 2.5 (-9.8-22.4) |  |  |
|  | 3 | 11 | 1.6 (-80.5-41.6) |  |  |
| StrideTimeSecLF | 1 | 20 | 0.6 (0.4-0.8) | 0.0692 | 0.1108 |
|  | 2 | 15 | 0.7 (0.5-0.9) |  |  |
|  | 3 | 11 | 0.5 (0.4-0.8) |  |  |
| StrideTimeSecLH | 1 | 20 | 0.6 (0.4-0.8) | 0.6658 | 0.6786 |
|  | 2 | 15 | 0.6 (0.5-1.1) |  |  |
|  | 3 | 11 | 0.7 (0.4-1.9) |  |  |
| StrideTimeSecRF | 1 | 20 | 0.6 (0.4-0.8) | 0.0625 | 0.1069 |
|  | 2 | 15 | 0.7 (0.5-1.0) |  |  |
|  | 3 | 11 | 0.5 (0.3-0.8) |  |  |
| StrideTimeSecRH | 1 | 20 | 0.6 (0.4-0.8) | 0.6911 | 0.6911 |
|  | 2 | 15 | 0.6 (0.5-1.2) |  |  |
|  | 3 | 11 | 0.7 (0.4-1.0) |  |  |
| StrideTime_LeftHind_RightHind | 1 | 20 | 1.5 (0.0-3.9) | 0.0000 | 0.0000 |
|  | 2 | 15 | 4.6 (1.1-62.5) |  |  |
|  | 3 | 11 | 8.1 (1.5-78.8) |  |  |
| StrideVelocityFrontHind | 1 | 20 | 2.6 (0.5-6.0) | 0.0340 | 0.0753 |
|  | 2 | 15 | 5.2 (1.4-31.6) |  |  |
|  | 3 | 11 | 2.6 (-7.4-44.2) |  |  |
| StrideVelocityLeft_HindRightHi | 1 | 20 | 1.9 (-2.7-11.2) | 0.0504 | 0.0890 |
|  | 2 | 15 | 4.0 (-4.2-51.3) |  |  |
|  | 3 | 11 | 3.1 (-99.2-92.9) |  |  |
| SwingTimeSecLF | 1 | 20 | 0.3 (0.2-0.3) | 0.1037 | 0.1381 |
|  | 2 | 15 | 0.3 (0.2-0.5) |  |  |
|  | 3 | 11 | 0.3 (0.2-0.5) |  |  |
| SwingTimeSecLH | 1 | 20 | 0.3 (0.2-0.3) | 0.0711 | 0.1108 |
|  | 2 | 15 | 0.3 (0.3-0.6) |  |  |
|  | 3 | 11 | 0.4 (0.3-1.4) |  |  |
| SwingTimeSecRF | 1 | 20 | 0.3 (0.2-0.3) | 0.1035 | 0.1381 |
|  | 2 | 15 | 0.3 (0.2-0.5) |  |  |
|  | 3 | 11 | 0.3 (0.2-0.5) |  |  |
| SwingTimeSecRH | 1 | 20 | 0.3 (0.2-0.4) | 0.0341 | 0.0753 |
|  | 2 | 15 | 0.3 (0.2-0.8) |  |  |
|  | 3 | 11 | 0.3 (0.2-0.7) |  |  |
| VIDLF | 1 | 20 | 30.2 (19.8-32.9) | 0.0998 | 0.1381 |
|  | 2 | 15 | 33.3 (23.8-37.2) |  |  |
|  | 3 | 11 | 31.6 (18.9-39.3) |  |  |
| VIDLH | 1 | 20 | 19.7 (11.2-22.8) | 0.2675 | 0.2847 |
|  | 2 | 15 | 17.6 (6.9-35.2) |  |  |
|  | 3 | 11 | 19.6 (6.4-25.5) |  |  |
| VIDRF | 1 | 20 | 30.4 (19.9-33.1) | 0.1028 | 0.1381 |
|  | 2 | 15 | 32.7 (20.0-43.4) |  |  |
|  | 3 | 11 | 29.6 (23.2-43.9) |  |  |
| VIDRH | 1 | 20 | 18.9 (12.2-22.5) | 0.0247 | 0.0623 |
|  | 2 | 15 | 16.2 (6.2-21.1) |  |  |
|  | 3 | 11 | 18.1 (12.5-27.2) |  |  |
| VIkgsecLF | 1 | 18 | 4.7 (0.4-18.9) | 0.0147 | 0.0433 |
|  | 2 | 14 | 3.9 (0.4-11.8) |  |  |
|  | 3 | 11 | 0.7 (0.1-6.6) |  |  |
| VIkgsecLH | 1 | 18 | 3.3 (0.3-11.5) | 0.0076 | 0.0336 |
|  | 2 | 14 | 2.1 (0.4-6.1) |  |  |
|  | 3 | 11 | 0.4 (0.1-3.9) |  |  |
| VIkgsecRF | 1 | 18 | 4.8 (0.5-19.5) | 0.0063 | 0.0336 |
|  | 2 | 14 | 4.2 (0.4-14.3) |  |  |
|  | 3 | 11 | 0.7 (0.1-5.5) |  |  |
| VIkgsecRH | 1 | 18 | 3.1 (0.2-12.3) | 0.0126 | 0.0433 |
|  | 2 | 14 | 1.9 (0.1-5.7) |  |  |
|  | 3 | 11 | 0.4 (0.0-3.5) |  |  |
| Weight | 1 | 20 | 22.4 (7.6-40.5) | 0.0122 | 0.0433 |
|  | 2 | 15 | 30.0 (17.0-59.0) |  |  |
|  | 3 | 10 | 16.5 (4.8-52.0) |  |  |
